# Effect of Different Carbon-Supported Catalysts on the Thermal Decomposition of Energetic Thermoplastic Elastomers

**DOI:** 10.3390/ma19081542

**Published:** 2026-04-12

**Authors:** Zhu Wang, Wenhao Liu, Haoyu Yu, Tianqi Li, Yunjun Luo, Yonghao Xiao

**Affiliations:** 1 School of Materials Science and Engineering, Beijing Institute of Technology, Beijing 100081, China; 2 AVIC Manufacturing Technology Institute, Beijing 100024, China; 3 Key Laboratory for Ministry of Education of High Energy Density Materials, Ministry of Education, Beijing Institute of Technology, Beijing 100081, China; 4 College of Light Industry Science and Engineering, Tianjin University of Science and Technology, Tianjin 300457, China

**Keywords:** GAP-ETPE, thermal decomposition, carbon-based composite catalyst

## Abstract

To enhance the thermal decomposition properties of glycidyl azide polymer energetic thermoplastic elastomer (GAP-ETPE), the effects of nano-CuO supported on different carbon carriers (GO and CNT) were systematically investigated in this study. The structural characteristics and catalytic performances were comprehensively analyzed using XRD, Raman, XPS, UPS, BET, SEM, and TEM, coupled with thermal analysis techniques including TG-DSC and TG-MS. The results indicate that the catalytic performance follows the descending order of CuO/CNT > CuO/GO > CuO. Notably, CuO/CNT exhibits the optimal catalytic activity, advancing the exothermic peak temperature of the azide groups by approximately 33 °C and resulting in a more concentrated heat release process. The superior synergistic catalytic effect of CuO/CNT is attributed to the following: the three-dimensional network constructed by CNT effectively overcomes the agglomeration of CuO nanoparticles and the restacking defects typical of GO nanosheets, thereby significantly reducing the gas–solid mass transfer resistance. Simultaneously, the highly graphitized sp^2^ conjugated skeleton of CNT provides an exceptional electron transport capability, facilitating rapid electron migration. These findings demonstrate that the structure of carbon supports profoundly influences the synergistic catalytic effect of CuO, offering valuable insights into the design of highly efficient catalysts for energetic binders.

## 1. Introduction

Energetic thermoplastic elastomers (ETPEs) have emerged as a vital development direction for solid propellant binders due to their unique combination of high energy, low sensitivity, low signature, and recyclability [1,2,3]. Among them, glycidyl azide polymer (GAP), a typical energetic prepolymer, is widely utilized as the soft segment in energetic binders owing to its high positive enthalpy of formation (approximately +957 kJ·kg^−1^), substantial gas yield upon decomposition, and excellent compatibility with nitrate ester plasticizers [4,5,6]. By employing specific diisocyanates and chain extenders as hard segments, the synthesized GAP-ETPE not only retains the high-energy characteristics of GAP but also endows the material with outstanding mechanical properties through its microphase-separated structure [7,8,9]. Although the decomposition of the -N_3_ groups in GAP-ETPE is a highly exothermic process, it occurs within a relatively high-temperature range, which may limit the combustion efficiency and burning rate of the overall propellant. Therefore, the introduction of highly efficient thermal decomposition catalysts to improve its thermal decomposition and combustion characteristics is a current research focus in this field.

Transition metal oxides have attracted extensive attention owing to their favorable catalytic activity [10]. Among them, copper oxide (CuO) has demonstrated particularly outstanding performance [11]. CuO is a typical p-type semiconductor possessing distinctive electronic structural characteristics [12,13,14]. The electron configuration of Cu^2+^ ions endows CuO with considerable redox flexibility; the Cu^2+^/Cu^+^ redox couple can alternately serve as an electron acceptor and donor during catalytic cycles, which is considered one of the key driving forces underlying the catalytic activity of CuO toward a variety of reactions [15]. The pendant azide groups (–CH_2_N_3_) on the side chains of GAP-ETPE feature a linear -N=N=N resonance structure, in which the non-uniform charge distribution imparts a partially weakened bond character to the Nα–Nβ linkage, rendering it the preferential cleavage site during thermal decomposition [16,17]. On the basis of transition-metal catalysis theory, it is postulated that the coordinatively unsaturated Cu^2+^ sites on the CuO surface interact with the azide moiety at the orbital level through a synergistic σ-coordination/π-back-donation mechanism, injecting electron density into the Nα–Nβ antibonding orbital, thereby weakening the N–N bond and lowering the decomposition activation energy. Concurrently, oxygen vacancy defect states generate Cu^+^ species that further enhance the efficiency of π-back-donation, augmenting the overall catalytic effect [18,19]. Recent studies have demonstrated that the catalytic activity of CuO and Cu_2_O nanostructures is highly dependent on their size and morphology; variations in quantum confinement effects and surface defect-state density significantly influence charge-carrier generation and transport, thereby modulating surface chemical reactivity [20,21]. Moreover, CuO nanoparticles exhibit a pronounced Mie scattering resonance effect, whose localized electromagnetic field enhancement is closely associated with energy coupling in catalytic reactions [22]. Investigations into the thermostructural properties of the Cu(II) complex [23] have further revealed the critical influence of the coordination environment on the electronic state of the Cu center and the thermal decomposition pathway. Collectively, these fundamental studies indicate that the performance of CuO-based catalysts is governed not only by their chemical composition but also by the electronic structure, defect chemistry, and interfacial interactions at the nanoscale.

However, nano-CuO is highly susceptible to agglomeration within polymeric matrices, which reduces the number of accessible catalytic active sites and severely limits catalytic efficacy. Loading nano-CuO onto carbonaceous supports to construct hybrid catalytic systems represents an effective strategy for mitigating agglomeration while simultaneously introducing synergistic catalytic effects [24,25]. Carbon materials, by virtue of their excellent electron-transport properties and tunable surface chemistry, have been widely employed to modulate the thermal decomposition behavior of polymers [26,27,28]. Surface functional groups on carbon materials can anchor CuO nanoparticles through the formation of Cu–O–C covalent bonds, whereas the highly conjugated sp^2^ electron network of carbon provides rapid pathways for interfacial charge transfer [29,30]. Nevertheless, different carbon supports exhibit intrinsic differences in electronic properties and heat-transfer characteristics: the van der Waals force between two-dimensional graphene oxide (GO) nanosheets tend to induce restacking, restricting reactant accessibility and interlayer electron transport [31,32]; in contrast, one-dimensional carbon nanotubes (CNT) establish three-dimensional open networks through their tubular architecture, offering superior electron-transport pathways, while the intertwined intertube arrangement creates a hierarchical pore structure that substantially reduces mass-transfer resistance [33,34]. Consequently, understanding the difference in catalytic behavior between CuO/GO and CuO/CNT systems from the perspective of semiconductor–carbon heterogeneous interfaces is of considerable scientific significance for establishing the structure–activity relationship linking “support dimensionality–interfacial coupling–catalytic activity.” In recent years, remarkable progress has been achieved in the application of Cu-based nanocatalysts to the thermal decomposition of energetic materials [11,35,36], and carbon nanomaterial-based composite catalytic systems have also demonstrated excellent catalytic performance in the decomposition of energetic components such as ammonium perchlorate (AP) [24,25,34,37,38,39]. These studies provide valuable references for understanding the dimensionality effect of carbon supports.

Based on the foregoing analysis, the following scientific hypothesis is proposed: carbon supports of different dimensionalities will exert differentiated influences on catalytic activity through distinct electron-transport and heat/mass-transfer efficiencies. To test this hypothesis, CuO/GO and CuO/CNT composite catalysts were prepared in this work. The crystal-phase structure, interfacial chemical states, specific surface area, and micromorphology of the catalysts were systematically characterized by X-ray diffraction (XRD), Raman spectroscopy, X-ray photoelectron spectroscopy (XPS), Brunauer–Emmett–Teller (BET) analysis, and scanning/transmission electron microscopy (SEM/TEM). The catalytic effects on the thermal decomposition behavior of GAP-ETPE were then systematically evaluated using thermogravimetry–differential scanning calorimetry (TG-DSC) and thermogravimetry–mass spectrometry (TG-MS). This study aims to establish, from a materials-chemistry perspective, the structure–activity relationship of “support dimensionality–microstructure–catalytic performance,” thereby providing experimental evidence and theoretical insights for the rational design of high-efficiency catalysts for energetic binders.

## 2. Experiments and Characterization

### 2.1. Materials

Copper(II) oxide nanoparticles (CuO) and copper(II) acetate monohydrate (Cu(CH_3_COO)_2_·H_2_O) were purchased from Shanghai (China) Macklin Biochemical Co., Ltd. Ethylene glycol and tetrahydrofuran (THF) were obtained from Shanghai (China) Aladdin Biochemical Technology Co., Ltd. Sodium hydroxide (NaOH) and 0.1 mol/L hydrochloric acid (HCl) were supplied by Tianjin (China) Fuchen Chemical Reagent Co., Ltd. and Tianjin (China) Damao Chemical Reagent Factory, respectively. All the aforementioned chemical reagents were of analytical grade (AR) and used as received without further purification. Graphene oxide (GO, thickness: 0.55–1.2 nm, lateral size: 0.5–3 μm, layers: <3, purity: >99 wt%) and carboxylated multi-walled carbon nanotubes (CNT, outer diameter: 5–15 nm, inner diameter: 2–5 nm, length: 10–30 μm, purity: >98 wt%) were provided by Tianjin (China) Zancheng Technology Co., Ltd. Distilled water was prepared in our laboratory. The glycidyl azide polymer energetic thermoplastic elastomer (GAP-ETPE) was synthesized in our laboratory according to the method described in the literature [40].

### 2.2. Synthesis of Carbon-Supported CuO/GO and CuO/CNT Catalysts

Carbon materials possess large specific surface areas, demonstrating considerable potential for nanoparticle loading applications. However, the weak interactions between pristine carbon materials and nanoparticles render them unsuitable for the in situ growth of nanoparticles. Therefore, graphene oxide (GO) bearing abundant carboxyl and hydroxyl groups, along with carboxylated CNT (CNT), were selected as carbon supports. The electronegative functional groups on these supports can serve as active sites to interact with nanoparticles through electrostatic forces and van der Waals interactions, thereby facilitating nanoparticle immobilization [41].

#### 2.2.1. Synthesis of CuO/GO Catalyst

The CuO/GO nanocomposites were prepared via an electrostatic self-assembly method. First, a certain mass of GO was dispersed in deionized water and ultrasonicated for 30 min to prepare a 1 mg/mL colloidal solution. Meanwhile, 0.3 g of CuO nanoparticles were dispersed in deionized water under ultrasonication for 30 min. The pH of the resulting suspension was adjusted to 3 using a 0.1 mol/L HCl solution, followed by its addition to 30 mL of the aforementioned GO solution. The mixture was further ultrasonicated for 2 h to ensure thorough mixing. Finally, the CuO/GO nanocomposites were obtained through filtration, washing, and drying. According to the thermogravimetric analysis (TGA) conducted in air atmosphere up to 800 °C (Figure S2), the actual CuO loading in the CuO/GO was determined to be 20.5 wt%.

#### 2.2.2. Synthesis of CuO/CNT Catalyst

The CuO/CNT nanocomposites were prepared via a sol-impregnation method at atmospheric pressure and a low temperature of 100 °C. First, 2.5 g of Cu(CH_3_COO)_2_·H_2_O and 1.0 g NaOH (with a molar ratio of 1:2) were separately dissolved in ethylene glycol under ultrasonication. The two solutions were then mixed to form a blue Cu(OH)_2_ sol, into which 0.5 g of CNT were immediately added to the sol. The mixture was heated to 100 °C under magnetic stirring, allowed to react for several hours, and subsequently filtered. Afterwards, 150 mL of distilled water was added to the obtained product, and the suspension was refluxed at 100 °C for 4 h. Finally, the CuO/CNT nanocomposites were obtained after washing, filtration, and drying. According to Figure S2, the CuO loading in the CuO/CNT was determined to be 17.6 wt%.

### 2.3. Preparation of GAP-ETPE Composites with Three Different Thermal Decomposition Catalysts

GAP-ETPE was dissolved in tetrahydrofuran (THF) in a flask under continuous stirring in a 60 °C oil bath to prepare a 0.1 g/mL solution. Subsequently, the thermal decomposition catalyst (CuO, CuO/GO, or CuO/CNT) was added at 5 wt% relative to the mass of GAP-ETPE. The mixture was pre-mixed via magnetic stirring for 1 h and then ultrasonicated in a water bath for 2 h to obtain a homogeneous composite dispersion. The dispersion was then cast into a polytetrafluoroethylene (PTFE) mold, left to evaporate at room temperature for 1 day, and vacuum-dried at 60 °C for 4 h to completely remove the solvent. Finally, the resulting black composite films were demolded and designated as CuO@ETPE, CuO/GO@ETPE, and CuO/CNT@ETPE, respectively.

### 2.4. Characterization

#### 2.4.1. Characterization of Thermal Decomposition Catalysts

The crystalline structures of the catalysts were characterized by X-ray diffraction (XRD, Bruker D8 ADVANCE, Karlsruhe, Germany) using Cu Kα radiation (λ = 1.54056 Å) over a 2*θ* scanning range of 10–80°. Raman spectra were recorded on a Raman spectrometer (Horiba Jobin Yvon HR-800, Longjumeau, France) to analyze the structural features of the samples, employing an excitation wavelength of 532 nm within a wavenumber range of 200–2000 cm^−1^. The surface elemental compositions and chemical states were analyzed via X-ray photoelectron spectroscopy (XPS, Thermo ESCALAB 250, Waltham, MA, USA) equipped with a monochromatic Al Kα X-ray source (hν = 1486.6 eV). All binding energies were calibrated referencing the adventitious C 1s peak at 284.8 eV. Ultraviolet photoelectron spectroscopy (UPS) measurements were performed on a photoelectron spectrometer (Thermo Fisher ESCALAB 250Xi, Waltham, MA, USA) employing a He I gas discharge lamp (hν = 21.22 eV) as the excitation source. To accurately determine the secondary electron cutoff (SECO), variable negative sample biases (0 V, −5 V, and −10 V) were applied to accelerate the low-energy secondary electrons. The specific surface areas and pore structures were evaluated by N_2_ adsorption–desorption isotherms measured at liquid nitrogen temperature (77 K) using a physisorption analyzer (Micromeritics TriStar II 3020, Norcross, GA, USA). The specific surface areas were calculated utilizing the Brunauer–Emmett–Teller (BET) method, while the pore size distributions were determined based on the Barrett–Joyner–Halenda (BJH) model. Furthermore, the microscopic morphologies of the samples were observed employing a field-emission scanning electron microscope (SEM, Hitachi Regulus 8230, Tokyo, Japan) and a transmission electron microscope (TEM, JEOL JEM-F200, Tokyo, Japan).

#### 2.4.2. Thermal Analysis

Analysis of thermal decomposition behavior: A simultaneous thermal analyzer (TGA-DSC, METTLER TOLEDO TGA/DSC 3+, Greifensee, Switzerland) was employed to evaluate the effects of the catalysts on the thermal decomposition characteristics of the samples. Approximately 0.5–1.0 mg of the sample was placed in an open ${\mathrm{Al}}_{2}O_{3}$ crucible. The tests were conducted from 50 °C to 500 °C at a heating rate of 10 °C/min under a high-purity argon atmosphere (flow rate: 40 mL/min). TG and DSC curves were recorded to determine the decomposition temperatures and heat release.

Analysis of gaseous products and mechanism: A thermogravimetry-mass spectrometry (TG-MS) coupling system (Netzsch STA 449 C-QMS 403 Aëolos, Selb, Germany) was employed to monitor the evolved gaseous products in real time during the thermal decomposition process to elucidate the catalytic mechanism. The TG test conditions were identical to those described above. The evolved gases were transferred into the mass spectrometer via a quartz capillary transfer line heated to 200 °C The mass spectrometer was operated with an electron ionization (EI) source at an ionization energy of 70 eV, and the mass scanning range was set to *m*/*z* = 10–200.

## 3. Results

### 3.1. Structural Characterization and Morphological Analysis of the Thermal Decomposition Catalysts

#### 3.1.1. Crystal Structure of the Thermal Decomposition Catalysts

**Figure 1:**
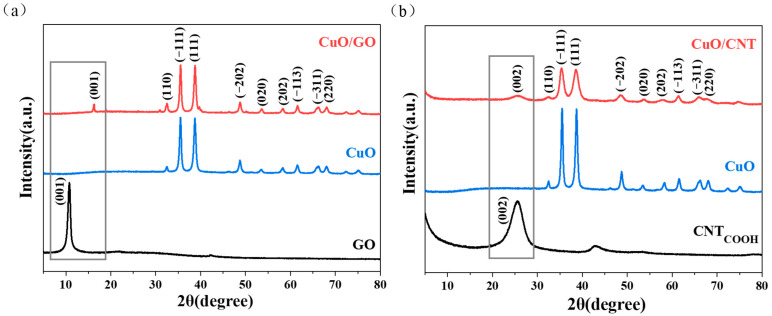
X-ray diffraction patterns of composite materials: (**a**) CuO/GO, (**b**) CuO/CNT.

Figure 1 shows the X-ray diffraction (XRD) patterns of the CuO/GO and CuO/CNT. Both composites exhibit distinct diffraction peaks at 2*θ* = 32.5°, 35.5°, 38.7°, 48.7°, 53.5°, 58.3°, 61.5°, 66.2°, and 68.0°, which correspond to the (110), (−111), (111), (−202), (020), (202), (−113), (−311), and (220) crystal planes of monoclinic CuO, respectively. These peaks are highly consistent with the standard pattern (JCPDS No. 45-0937), indicating that CuO exists in a monoclinic crystalline phase in both composites.

As shown in Figure 1a, the characteristic (001) diffraction peak of GO in CuO/GO shifts from 10.78° to 16.36°. According to Bragg’s law, the corresponding interlayer spacing (*d*-spacing) decreases from 0.82 nm to 0.54 nm. This change indicates that GO is partially reduced during the preparation of the composite, and the removal of oxygen-containing functional groups leads to the collapse of the graphitic layers [42,43], which is further corroborated by the subsequent XPS analysis. The reduction in *d*-spacing facilitates enhanced electronic interactions between GO and the CuO particles, thereby improving the electrical conductivity of the composite. In Figure 1b, pure CNT exhibit a broad peak around 2*θ* ≈ 26°, corresponding to the (002) crystal plane of the graphitic structure, which reflects its typical multi-walled tubular characteristics.

Comparing the diffraction peak profiles of CuO in the two composites, the characteristic peaks of CuO in CuO/GO are sharper with higher intensity, indicating a higher crystallinity and a larger crystallite size. Conversely, the corresponding peaks in CuO/CNT are significantly broadened, suggesting a smaller crystallite size and better dispersion of CuO. Owing to the distinct surface chemistries of GO and CNT, CuO/GO and CuO/CNT were synthesized via electrostatic self-assembly and sol-impregnation methods, respectively. This methodological discrepancy may partially account for the variations in CuO crystallite size. As indicated by XRD peak broadening analysis, CuO nanoparticles on CNT exhibit smaller crystallite sizes and higher dispersion uniformity compared to those on GO, which can be partially attributed to the in situ nucleation and growth mechanism inherent in the sol-impregnation process. Furthermore, the intrinsic structural properties of the carbon supports also play a critical role: the three-dimensional network formed by intertwined 1D CNTs exerts a stronger steric hindrance effect, effectively inhibiting the excessive growth and agglomeration of CuO nuclei. Conversely, 2D GO nanosheets are prone to π-π stacking, which weakens the confinement effect on CuO grain growth. As reported in related research [21], the size of CuO nanoparticles directly regulates their catalytic activity. Consequently, the variations in CuO crystallite sizes between CuO/GO and CuO/CNT may lead to distinct catalytic performances.

#### 3.1.2. Molecular Structural Features of the Thermal Decomposition Catalysts

To further investigate the lattice vibration modes, microstructural ordering, and interfacial interactions between CuO and the carbon supports, Raman spectroscopy was performed on CuO, GO, CNTs, and the prepared CuO/GO and CuO/CNT composites [44,45].

**Figure 2:**
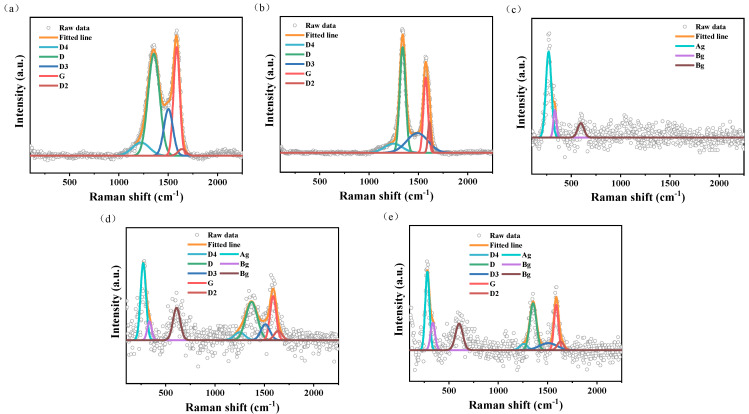
Raman spectroscopy: (**a**) GO, (**b**) CNT, (**c**) CuO, (**d**) CuO/GO, (**e**) CuO/CNT.

As shown in Figure 2a,b, both GO and CNT exhibit the typical dual-band features of carbon-based materials: the D band (defect band) at ~1350 cm^−1^ and the G band (graphitic band) at ~1590 cm^−1^ [46]. Pure CuO displays distinct characteristic peaks at 290 cm^−1^, 340 cm^−1^, and 630 cm^−1^, which are assigned to the A_g_ vibrational mode (symmetric stretching of the Cu-O bond along the b-axis) and B_g_ vibrational modes (antisymmetric bending and stretching) of monoclinic CuO, respectively [47,48]. These results corroborate the aforementioned XRD phase analysis.

Figure 2d,e present the Raman spectra of the composites. The spectra of CuO/GO and CuO/CNT contain the characteristic peaks of both the carbon supports and CuO, confirming their successful integration. By comparing the defect densities (intensity ratio $I_{D}/I_{G}$), it is observed that after CuO loading, the $I_{D}/I_{G}$ values of CuO/GO and CuO/CNT increase from 1.71 and 1.14 to 1.82 and 1.52, respectively. This increase indicates that the nucleation and growth of CuO particles on the carbon surface disrupt a portion of the sp^2^ conjugated network, leading to an increase in local structural disorder.

Compared to the individual components, the D and G bands of the carbon supports, as well as the A_g_ and B_g_ modes of CuO in the composites, all exhibit a distinct blue shift. The shift in Raman peak positions in the composite system may originate from multiple factors, including lattice strain effects, the introduction of defect states, and charge transfer between the components. Regarding the carbon supports, the nucleation and growth of CuO nanoparticles on the carbon surface impose compressive strain on the local carbon skeleton and simultaneously disrupt a portion of the sp^2^ conjugated network (evidenced by the increased $I_{D}/I_{G}$ ratio), both of which can lead to the blue shift in the D and G bands. Furthermore, since the work function of the p-type semiconductor CuO is lower than that of the carbon materials [49,50,51], electrons can spontaneously transfer from the carbon supports to CuO upon interfacial contact. The loss of electrons from the carbon materials weakens the electron shielding effect of their π−π conjugated system, thereby increasing the C−C bond force constant and consequently triggering the Raman shift. For CuO, the blue shift in its A_g_ and B_g_ modes can be attributed to the compressive strain imposed on the CuO lattice by the rigid network of the carbon supports, resulting in shortened Cu-O bond lengths and increased vibrational frequencies. Simultaneously, the variation in the electron density of CuO induced by interfacial charge transfer may also modulate its bond vibrational frequencies.

Additionally, in the composites, the A_g_ peak of CuO undergoes sharpening, while the B_g_ peak exhibits broadening. It is speculated that during the composite synthesis process, the oxygen-containing functional groups and topological structures on the carbon support surfaces serve as heterogeneous nucleation sites, which to some extent induce the oriented growth of CuO and enhance the internal structural ordering of the crystallites, manifesting as the sharpening of the A_g_ peak. Concurrently, the strong interfacial interaction between CuO and the carbon supports may disrupt the lattice symmetry on the CuO surface. The resulting increase in the interfacial disordered layer exacerbates the inhomogeneous broadening of the B_g_ vibrational mode, leading to its broadened peak shape. The differential response of the A_g_ and B_g_ peak profiles further indicates that the interaction between CuO and the carbon supports is not a simple physical mixing, but involves structural and electronic coupling at the interfacial level.

In summary, Raman spectroscopic analysis confirms the successful loading of CuO onto the carbon supports and reveals the existence of significant interfacial interactions between them. The shifts in characteristic peaks and the variation in the $I_{D}/I_{G}$ ratio in the composites reflect the superposition of multiple effects, including strain, defects, and charge transfer. Although it is challenging to individually quantify the contribution of charge transfer solely through Raman spectroscopy, combining these results with the subsequent XPS analysis—specifically the shift in Cu 2p binding energy and the variation in the satellite peak intensity ratio ($I_{sat}/I_{main}$)—can more reliably confirm the presence of the charge transfer effect between CuO and the carbon supports. This interfacial coupling is expected to provide pathways for rapid electron transport at the interface, which is closely correlated with the excellent performance exhibited by the composite catalysts in the subsequent catalytic thermal decomposition of GAP-ETPE.

#### 3.1.3. Surface Chemical States and Elemental Composition of the Thermal Decomposition Catalysts

**Figure 3:**
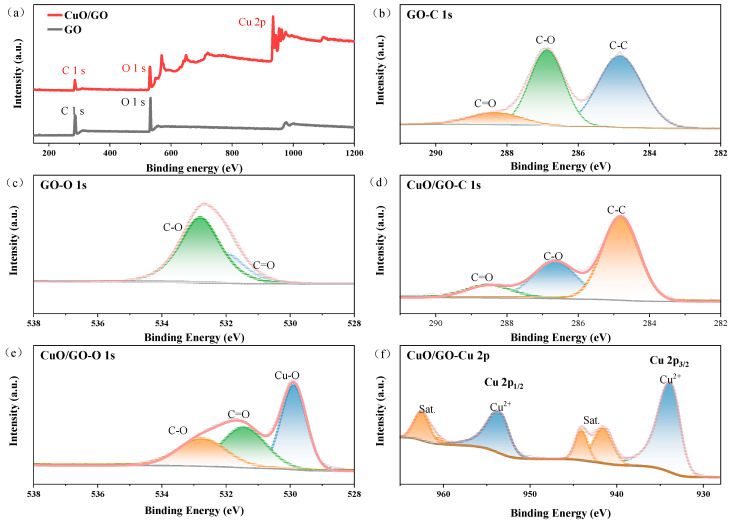
XPS spectra of GO and CuO/GO: (**a**) survey spectra; high-resolution (**b**) C 1s and (**c**) O 1s spectra of GO; high-resolution (**d**) C 1s, (**e**) O 1s, and (**f**) Cu 2p spectra of CuO/GO.

**Figure 4:**
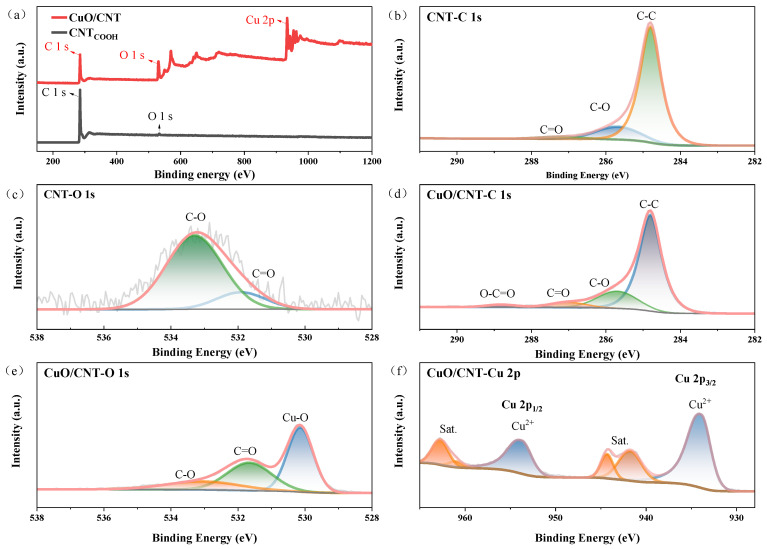
XPS spectra of CNT and CuO/CNT: (**a**) survey spectra; high-resolution (**b**) C 1s and (**c**) O 1s spectra of CNT; high-resolution (**d**) C 1s, (**e**) O 1s, and (**f**) Cu 2p spectra of CuO/CNT.

Figure 3a and Figure 4a present the XPS survey spectra of GO, CuO/GO and CNT, CuO/CNT, respectively. The emergence of the Cu 2p characteristic peak in the composites indicates the successful loading of CuO onto the carbon support surfaces. As determined by XPS analysis, the C and O contents in GO are 68.2% and 31.8%, respectively, while those in carboxylated CNT are 98.1% and 1.9%. For the composites, the C, O, and Cu contents in CuO/GO are 39.6%, 36.9%, and 23.5%, respectively, whereas those in CuO/CNT are 59.6%, 21.2%, and 19.2%, respectively.

Figure 3b,c and Figure 4b,c display the high-resolution C 1s and O 1s spectra of GO and CNT, respectively. GO exhibits a relatively stronger O 1s peak, indicating a higher abundance of surface oxygen containing functional groups. In contrast, the C-C main peak of CNT at ~284.6 eV is sharper and more intense, suggesting the retention of a highly crystalline wall structure. Meanwhile, the weak peaks in the ~285–288 eV range confirm the introduction of carboxyl (-COOH) groups at surface defect sites, which provide binding sites for the subsequent anchoring of nanoparticles.

Figure 3d–f and Figure 4d–f show the high-resolution C 1s, O 1s, and Cu 2p spectra of the composites, respectively. The O 1s spectra of the composites can be deconvoluted into Cu-O, C=O, and C-O bonds [52], confirming the formation of a Cu-O-C interfacial structure. This indicates a chemical coordination, rather than a mere physical mixture, between CuO and the oxygen containing groups on the carbon supports. Furthermore, the relative intensities of the oxygenated carbon bonds (C-O and C=O) in CuO/GO are significantly lower than those in pure GO, demonstrating that GO underwent a certain degree of reduction during the electrostatic self-assembly process [25], which is consistent with the XRD deduction. In the Cu 2p spectra, the Cu 2p_3_/_2_ and Cu 2p_1_/_2_ peaks are located at ~933–934 eV and ~953–954 eV, respectively, accompanied by distinct shake-up satellite peaks at ~941–944 eV and ~961–964 eV. These characteristic peaks are typical fingerprints of Cu^2+^, indicating that copper in the as-prepared composites predominantly exists in the Cu(II) chemical state.

To investigate the interfacial electronic interactions between CuO and the carbon supports, the high-resolution Cu 2p spectra of CuO/GO and CuO/CNT were analyzed. The binding energies of Cu 2p_3_/_2_ in CuO/GO and CuO/CNT are 933.6 eV and 933.5 eV, respectively, both exhibiting a negative shift compared to the reported reference value for pure CuO (~933.7 eV) [53], with the shift being more pronounced in CuO/CNT. Concurrently, both composites exhibit the characteristic satellite peaks of Cu^2+^. However, their intensity ratios of the satellite peak to the main peak ($I_{sat}/I_{main}$) are 0.35 and 0.32, respectively, which are significantly lower than the literature reference values for pure CuO (~0.45–0.55) [54], with CuO/CNT showing a lower ratio than CuO/GO. The negative shift in binding energy and the decreased relative intensity of the satellite peaks typically indicate an increased electron cloud density around CuO. This suggests a potential interfacial charge transfer from the carbon supports to CuO, wherein CNT exerts a stronger electron-donating effect. Furthermore, the detection of the Cu–O–C component in the O 1s spectra indicates the formation of interfacial chemical bonds within the composites, providing a structural foundation for electronic coupling and charge transfer.

#### 3.1.4. Ultraviolet Photoelectron Spectroscopy (UPS) Analysis of Interfacial Electronic Structures

**Figure 5:**
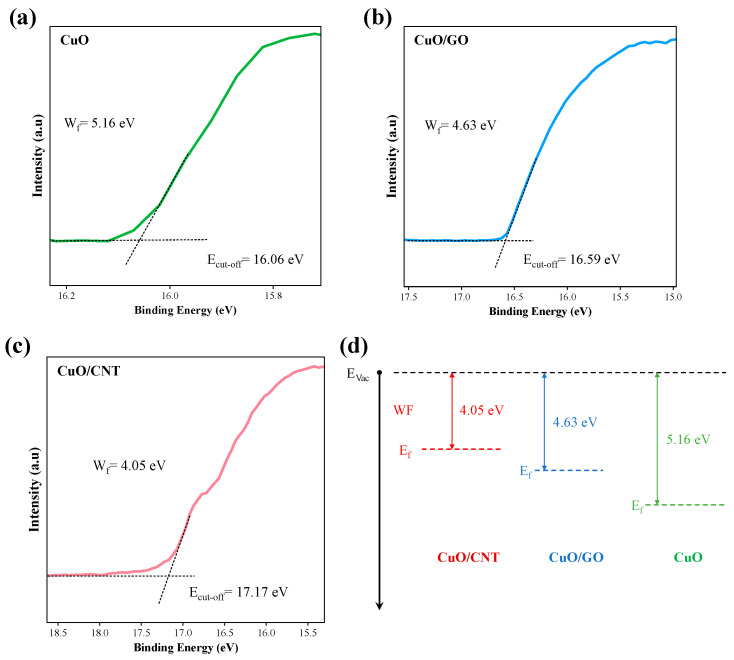
Work functions and Fermi level positions of CuO, CuO/GO, and CuO/CNT derived from ultraviolet photoelectron spectroscopy (UPS) measurements: UPS secondary electron cutoff spectra of (**a**) CuO, (**b**) CuO/GO, and (**c**) CuO/CNT; (**d**) schematic diagram of the Fermi level positions.

To further investigate the interfacial electronic interactions within the catalysts, ultraviolet photoelectron spectroscopy (UPS) measurements were conducted on the samples, as illustrated in Figure 5. Based on the linear extrapolation of the secondary electron cutoff edge, the work functions of pure CuO, CuO/GO, and CuO/CNT are determined to be 5.16 eV, 4.63 eV, and 4.05 eV, respectively. Compared to pure CuO, the work functions of the composites decrease significantly upon the introduction of carbon supports. The most pronounced reduction is observed in CuO/CNT, indicating that the CNT support exerts a stronger modulating effect on the surface electronic structure of CuO. This structural modulation endows the CuO active sites in CuO/CNT with an enhanced electron-donating capacity. A lower work function signifies a reduction in the energy required for electrons to escape from the material surface, concurrently reflecting an upward shift in the Fermi level relative to the vacuum level. This demonstrates that a significant electronic structure reconstruction occurs at the interface following the integration of CuO with carbon supports, thereby facilitating interfacial electron redistribution and the enhancement of catalytic performance. It is worth noting that the aforementioned UPS data represent static characterization results, reflecting the equilibrium electronic structure of the as-prepared catalysts rather than the dynamic charge transfer behavior during the actual catalytic reaction process.

### 3.2. Pore Structure and Micromorphology of Thermal Decomposition Catalysts

#### 3.2.1. Specific Surface Area and Pore Structure of Thermal Decomposition Catalysts

**Figure 6:**
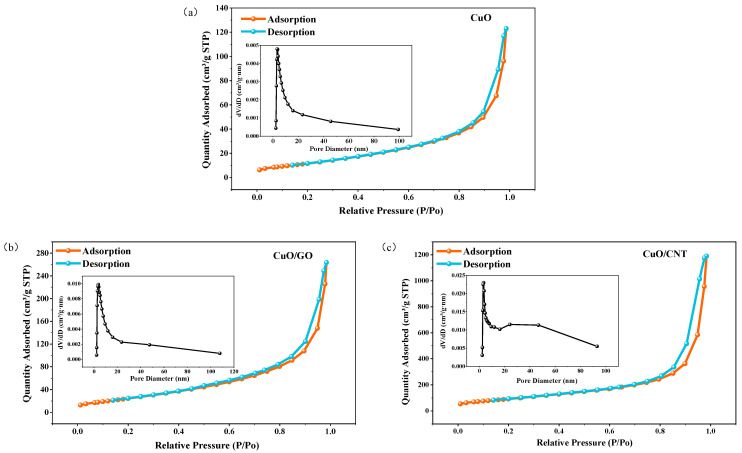
N_2_ adsorption–desorption isotherms and the corresponding pore size distribution curves: (**a**) CuO, (**b**) CuO/GO, and (**c**) CuO/CNT.

The pore structure parameters of materials are closely correlated with their catalytic performance. To investigate the regulatory effect of carbon-based supports on the pore structure, the specific surface area and pore characteristics of pure CuO and its composites (CuO/GO and CuO/CNT) were characterized using N_2_ adsorption–desorption measurements, as illustrated in Figure 6 and Table 1.

As depicted in Figure 6, all three samples exhibit distinct hysteresis loops in the relative pressure ($P/P_{0}$) range of 0.4–1.0, which is a typical characteristic of mesoporous structures. According to the IUPAC classification, all of them display Type IV isotherms accompanied by H3-type hysteresis loops. The area and width of the hysteresis loops vary significantly among the three samples: pure CuO presents the smallest hysteresis loop ($P/P_{0}$ = 0.85–0.95), CuO/GO shows a moderate one ($P/P_{0}$ = 0.80–0.92), while CuO/CNT exhibits the largest hysteresis loop ($P/P_{0}$ = 0.75–0.95). This indicates that the introduction of the CNT support generates a more complex pore structure.

Based on the BJH pore size distribution curves shown in the inset of Figure 6, the pore sizes of all three samples are concentrated in the small mesoporous range of 2–5 nm. Compared with pure CuO, the pore size distribution profile of CuO/GO remains essentially unchanged, but its differential pore volume peak (dV/dD) is elevated, suggesting that the two-dimensional GO nanosheets effectively increase the number of fine mesopores. For CuO/CNT, the dV/dD value is further enhanced, and a distinct secondary mesopore distribution band emerges in the range of 20–50 nm. This demonstrates that the introduction of CNT not only increases the total pore volume but also constructs a hierarchical pore system integrating both small and larger mesopores.

According to the pore structure parameters in Table 1, both parameters exhibit a consistent increasing trend. The specific surface area of CuO/GO is approximately 2.1 times that of pure CuO. After the introduction of CNT, the specific surface area is further boosted to 7.6 times that of CuO, and the total pore volume is enlarged by about 9.2 times. The substantial enhancement in the pore structure parameters of CuO/CNT can be attributed to two main factors: First, CNT possess a tubular structure with a high aspect ratio, which intertwine to form a three-dimensional network, effectively inhibiting the agglomeration of CuO nanoparticles. Second, compared to the possible π-π stacking between GO nanosheets, the one-dimensional structure of CNT is more effective in dispersing the active components, thereby forming larger-scale mesoporous channels.

In summary, the dimensionality and network configuration of the carbon supports exert a significant regulatory effect on the pore structure of the composites. Compared to two-dimensional GO, one-dimensional CNT are more capable of endowing the material with superior structural characteristics of “high specific surface area, high pore volume, and hierarchical pore sizes”. On the one hand, this structure significantly increases the number of surface active sites, which is conducive to promoting electron transfer during the catalytic reaction process. On the other hand, the open hierarchical pore channels, particularly the distinct secondary mesopore distribution band observed in CuO/CNT (20–50 nm), are crucial for facilitating the efficient diffusion and mass transfer of gaseous reactants and products during the thermal decomposition of the polymeric energetic binder, thereby significantly reducing gas–solid mass transfer resistance and enhancing the overall catalytic efficiency [55,56]. This structural advantage provides a crucial foundation for the subsequent enhancement of the catalytic performance of the CuO/CNT composites.

#### 3.2.2. Micromorphology of Thermal Decomposition Catalysts

**Figure 7:**
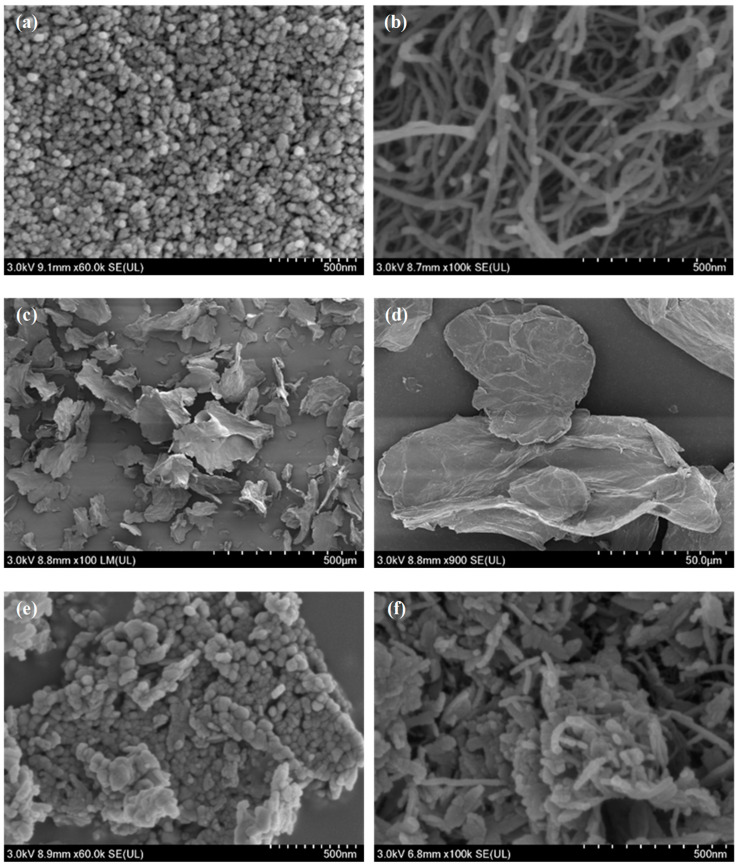
SEM images of different samples: (**a**) CuO, (**b**) CNT, (**c**,**d**) GO, (**e**) CuO/GO, (**f**) CuO/CNT.

To investigate the influence of carbon supports on the microscopic morphology and dispersion of CuO, scanning electron microscopy (SEM) was employed to characterize CuO, GO, CNT, and their composites, as shown in Figure 7. Pure CuO consists of agglomerated and stacked quasi-spherical nanoparticles, exhibiting a relatively dense structure with indistinct pores. GO presents a wrinkled two-dimensional (2D) nanosheet structure [57,58] with a certain degree of stacking. Conversely, CNT display an intertwined tubular network with a relatively open three-dimensional (3D) skeleton. After compounding, CuO nanoparticles are successfully loaded onto the surfaces of both the GO nanosheets and the CNT networks. Specifically, the dispersion of CuO particles in CuO/GO is improved; however, localized particle agglomeration persists due to the overlapping of GO nanosheets. In contrast, CuO particles in CuO/CNT are more uniformly distributed on the CNT surfaces, and the intact 3D network structure of CNT is preserved. This structural integrity facilitates the construction of open pore channels and continuous mass transfer pathways, which is consistent with its higher specific surface area and pore volume observed in the BET results.

To evaluate the regulatory effect of carbon supports on the dispersion state of CuO, statistical particle size analysis of CuO nanoparticles in pure CuO, CuO/GO, and CuO/CNT was conducted based on SEM images. The corresponding statistical results are presented in Figure S4 of the Supporting Information.

Under the preparation conditions employed in this study, the average particle size of CuO nanoparticles in CuO/CNT is 20.21 nm with a relatively narrow distribution curve, indicating excellent intra-sample uniformity. In contrast, the CuO nanoparticles in CuO/GO exhibit a larger average particle size of 24.39 nm and a broader distribution, reflecting the localized agglomeration and size inhomogeneity induced by the stacking of GO nanosheets. These results further demonstrate that the tubular network structure of one-dimensional (1D) CNTs effectively inhibits the agglomeration and excessive growth of CuO nanoparticles via strong steric hindrance, whereas the stacking of two-dimensional (2D) GO nanosheets weakens their confinement effect on CuO grain growth.

**Figure 8:**
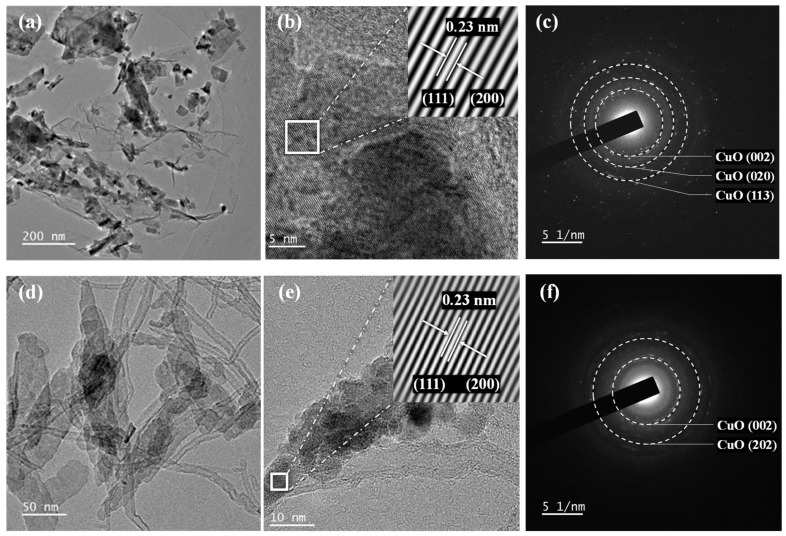
TEM, HRTEM, and SAED images of different samples: (**a**) TEM image of CuO/GO, (**b**) HRTEM image of CuO/GO, (**c**) SAED pattern of CuO/GO, (**d**) TEM image of CuO/CNT, (**e**) HRTEM image of CuO/CNT, (**f**) SAED pattern of CuO/CNT.

The internal structures of the composites were further analyzed using transmission electron microscopy (TEM), high-resolution TEM (HRTEM), and selected area electron diffraction (SAED). The results are shown in Figure 8.

In the HRTEM images of both composites, clear and continuous lattice fringes with an interplanar spacing of approximately 0.23 nm are observed, corresponding to the (111) or (200) characteristic crystal planes of monoclinic CuO. This indicates that the compounding process does not disrupt the crystalline structure of CuO. Furthermore, the SAED patterns exhibit clear concentric diffraction rings, demonstrating the typical polycrystalline nature of the loaded CuO, and no impurity diffraction rings are observed. These findings prove that CuO retains a high-purity monoclinic phase structure in both composite systems.

The TEM-EDS elemental mapping presented in Figure S1 clearly reveals the spatial distribution of C K, O K, and Cu K signals. The distribution of the Cu signal corresponds well with the locations of nanoparticles observed in the TEM bright-field image, confirming the successful deposition of CuO nanoparticles onto the carbon support surface. The EDS mapping results indicate a degree of inhomogeneity in the Cu signal distribution, with slight agglomeration observed in localized regions; nevertheless, the CuO nanoparticles remain predominantly dispersed at the nanoscale.

### 3.3. Effect of Thermal Decomposition Catalysts on the Thermal Decomposition Behavior of GAP-ETPE

**Figure 9:**
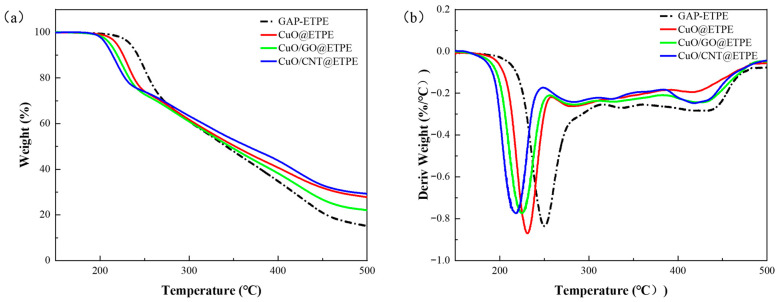
TG and DTG curves of GAP-ETPE and GAP-ETPE containing three combustion catalysts: (**a**) TG, (**b**) DTG.

**Figure 10:**
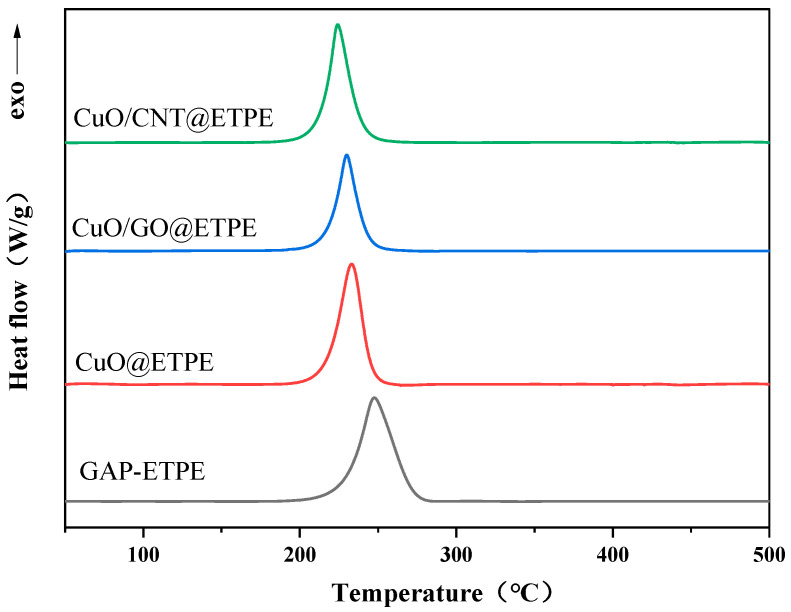
DSC curves of GAP-ETPE and GAP-ETPE containing three combustion catalysts.

As a crucial energetic binder in solid propellants, the thermal decomposition temperature of GAP-ETPE directly dictates the combustion performance of the system [59,60,61]. In this study, the regulatory effects of CuO, CuO/GO, and CuO/CNT on the thermal decomposition behavior of GAP-ETPE were systematically evaluated using coupled TG-DSC techniques (Figure 9 and Figure 10, and Table 2). The results indicate that the mass loss curves of the four samples exhibit highly consistent trends, all displaying typical four-stage decomposition characteristics. This indicates that, at the level of macroscopic decomposition pathways, the introduction of the catalyst does not alter the inherent four-stage pyrolysis characteristics of GAP-ETPE or the primary chemical processes involved in each stage. The catalyst functions primarily at the kinetic level by lowering the activation energy barrier of the rate-determining step, thereby promoting thermal decomposition at reduced temperatures without introducing novel reaction pathways.

The mass loss in the first stage is approximately 30%, which is highly consistent with the theoretical mass fraction (29.7%) of the pendant azide groups (–N_3_) in GAP-ETPE. In this stage, the maximum-mass-loss-rate peak ($T_{p1}$) on the DTG curve is located closely to the main exothermic peak ($T_{p}$) on the DSC curve (the DSC peak temperature is slightly higher due to the thermal hysteresis effect [62]), indicating that the mass loss originates from the intense exothermic decomposition of the –N_3_ groups. The subsequent three mass loss stages correspond to the decomposition of the urethane hard segments, the polyether soft segment backbone, and the residual skeleton, respectively [40]. Comparative thermal analysis results reveal that all three catalysts exhibit a significant promoting effect on the decomposition of the –N_3_ groups, shifting the characteristic decomposition peaks substantially toward the lower temperature region. Meanwhile, provided that the macroscopic pyrolysis mechanism remains unaltered, the apparent exothermic effect of the system shows no significant fluctuation. Furthermore, influenced by the combined effects of the catalytic carbonization of CuO [63] and the intrinsic thermal stability of the carbon supports, the char yields of CuO@ETPE, CuO/GO@ETPE, and CuO/CNT@ETPE at 500 °C are 27.8%, 22.1%, and 29.3%, respectively.

To rigorously evaluate the relative catalytic efficiency of the catalysts, non-isothermal kinetic analysis was conducted using the standard Kissinger method. The corresponding linear fitting plots and summarized kinetic parameters are provided in the Supporting Information (Figure S3 and Table S1). The results demonstrate that the apparent activation energies ($E_{a}$) of the GAP-ETPE, CuO/GO@ETPE, and CuO/CNT@ETPE systems are 162.41 kJ/mol, 148.09 kJ/mol, and 142.17 kJ/mol, respectively. The introduction of the composite catalysts significantly reduces the activation energy barrier for the decomposition of azide groups. Notably, the CuO/CNT@ETPE system exhibits the lowest $E_{a}$ value, which is 20.24 kJ/mol lower than that of the pure matrix.

It should be noted that differences in the preparation method may to some extent lead to variations between the two composites in terms of CuO grain size, sample homogeneity, and interfacial bonding quality. XRD analysis and SEM/TEM morphological characterization reveal that CuO nanoparticles supported on CNT possess smaller particle sizes and better sample homogeneity than those on GO. This can be partly attributed to the in situ nucleation and growth mechanism involved in the sol-impregnation method. Given the significant differences in the type and density of surface functional groups on the two carbon supports, it is practically challenging to synthesize both composites using an identical preparation procedure. Therefore, the comparative analysis in this work was performed under individually optimized preparation conditions with similar CuO loadings.

Overall, the catalytic efficiency decreases in the order: CuO/CNT > CuO/GO > CuO. The introduction of carbon supports not only effectively improves the dispersion of CuO, but their excellent electrical conductivity also accelerates electron transfer during the reaction and suppresses electron–hole recombination [64]. Among them, the CuO/CNT composite system exhibits the optimal synergistic catalytic activity.

### 3.4. Gaseous Products and Mechanism of Thermal Decomposition

**Figure 11:**
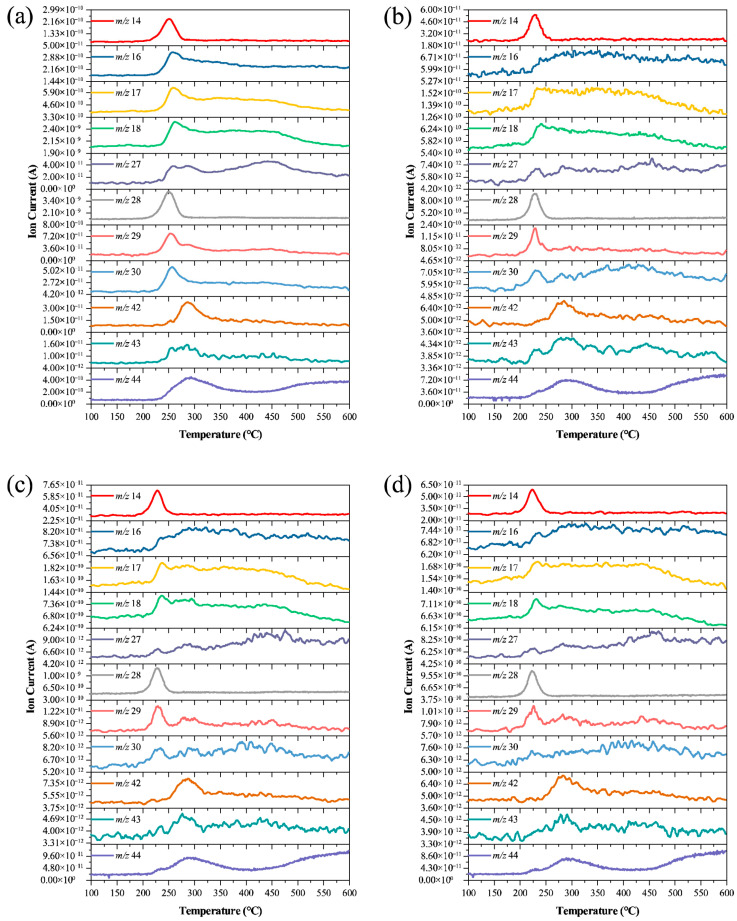
MS curves of gas products during decomposition at different temperatures: (**a**) GAP-ETPE, (**b**) CuO@ETPE, (**c**) CuO/GO@ETPE, (**d**) CuO/CNT@ETPE.

TG-MS measurements were performed on GAP-ETPE and catalyst@GAP-ETPE composite samples, and the results are shown in Figure 11. Ion signals with mass-to-charge ratios (*m*/*z*) of 14, 16, 17, 18, 27, 28, 29, 30, 42, 43, and 44 were detected in the thermal decomposition products of GAP-ETPE. This indicates the generation of N, O/NH_2_, OH/NH_3_, H_2_O, HCN, N_2_, CHO, CH_2_O, N_3_, HN_3_, and N_2_O/CO_2_ during the pyrolysis process [65]. Upon the addition of catalysts, identical ion signals to those of pristine GAP-ETPE were observed, demonstrating that the incorporation of catalysts does not alter the gaseous products of the thermal decomposition. However, the emergence time of the ion peaks (*m*/*z* = 14/28) corresponding to the N_2_ generated from the decomposition of azide groups is significantly advanced. This phenomenon is particularly pronounced for the CuO/CNT system, reflecting the highly efficient catalytic activity of the carbon-supported catalysts.

The above TG-MS results indicate that the introduction of catalysts does not lead to the emergence of new gaseous products or the disappearance of existing species, implying that the primary thermal decomposition pathway of GAP-ETPE remains unchanged at the macroscopic chemical level. The significant decrease in decomposition temperature and the advanced evolution time of gases essentially reflect the regulation of reaction kinetic parameters by the catalysts: the coordinatively unsaturated Cu(II) sites on the CuO surface lower the activation energy barrier for N–N bond cleavage through orbital interactions with the azide groups, enabling the bond scission to occur at lower temperatures. The more pronounced catalytic effect observed in the CuO/CNT system is attributed to the increased number of active sites and the excellent electron-transport properties of the CNT support.

Based on the relevant literature and the TG-MS analysis, the thermal decomposition mechanism of GAP-ETPE can be hypothesized as follows: The initial stage of pyrolysis corresponds to the cleavage of the pendant azide groups. Following the decomposition of the azide groups, the system tends to form a partially cross-linked or network structure, which subsequently suppresses the escape of small-molecule volatile products [66]. Consequently, only minor amounts of gases are released during the subsequent thermal degradation stages. During the first mass loss stage, based on the detected gaseous products, the decomposition of the pendant –CH_2_N_3_ groups is consistent with two possible pathways previously proposed in the literature [9,67]. The first pathway involves the cleavage of the R–N–N_2_ bond to release N_2_ (a portion of which may be converted to N_2_O in subsequent processes), accompanied by the formation of a nitrene intermediate. This intermediate further rearranges to form an imine group (C=N), which can subsequently undergo proton transfer and free-radical reactions to yield NH_3_, or undergo further scission to generate HCN. The second pathway is the direct scission of the R–N_3_ bond to generate an azide radical (N_3_·), which can further react with active hydrogen atoms or other free radicals to form HN_3_. The gaseous products detected in the second stage, such as CO_2_ and CH_2_O, primarily originate from the further degradation of the urethane groups (–NHCOO–) and the polyether soft segment backbone [11,68].

However, it is worth noting that the temporal resolution and sensitivity of TG-MS are limited, precluding the capture of the coordination environment on the catalyst surface and the lifetimes of transient intermediates. Therefore, the conclusion that the decomposition mechanism remains unaltered in this study should be interpreted as follows: within the current experimentally detectable range, the primary decomposition pathways and the species of gaseous products have not changed, rather than completely ruling out all possible subtle mechanistic differences.

### 3.5. Catalytic Mechanism

**Figure 12:**
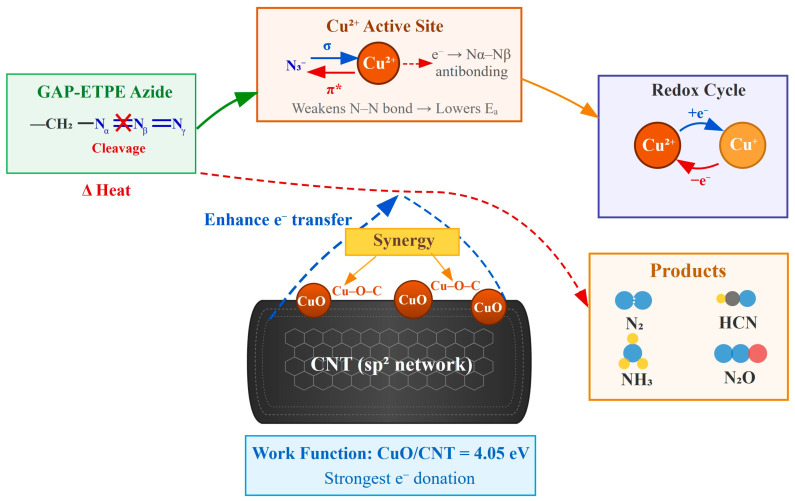
Schematic diagram of the catalytic mechanism.

To elucidate the regulatory mechanism of the carbon support type on the catalytic performance of CuO and the intrinsic reasons for the catalytic discrepancy between CuO/GO and CuO/CNT, this section comprehensively analyzes the catalytic mechanism based on the aforementioned characterization results. As shown in Figure 12.

First, from the perspective of the interfacial electronic structure, the UPS results reveal that the work functions of CuO, CuO/GO, and CuO/CNT are 5.16 eV, 4.63 eV, and 4.05 eV, respectively. The introduction of carbon supports significantly reduces the work function of the composites, with CuO/CNT exhibiting the most substantial decrease. This indicates the occurrence of electronic structure reconstruction at the interface after the hybridization of CuO with the carbon supports. Compared to GO, CNT exerts a more profound regulatory effect on the electronic structure of CuO, endowing the CuO active sites with a superior electron-donating capacity, which facilitates the catalytic reaction. It should be noted that the UPS data reflect the static electronic structure of the as-prepared samples; thus, the above analysis serves to illustrate the variation trend of the interfacial electronic environment rather than directly characterizing the transient charge transfer behavior during the thermal decomposition process.

Second, combining the thermal analysis results with relevant literature, the catalytic active sites in this system can be rationally deduced. The TG-DSC results demonstrate that pure CuO nanoparticles can also significantly promote the initial thermal decomposition of GAP-ETPE, advancing the exothermic decomposition peak. This implies that CuO itself possesses the crucial active sites for activating the azide groups. According to the conclusions of literature [15], in the Cu(II) coordination system, coordinatively unsaturated Cu(II) sites serve as the core active centers for the catalytic conversion of nitrogen-containing groups, and their local coordination environment decisively impacts the catalytic efficiency. Therefore, it can be reasonably inferred that the coordinatively unsaturated Cu(II) sites exposed on the surface of highly dispersed CuO are the core active sites responsible for catalyzing the decomposition of the pendant azide groups of GAP-ETPE in this system. Due to their coordinative unsaturation, these sites possess strong Lewis acidity and electron accepting/back-donating capabilities, which may strongly interact with the azide groups, thereby promoting the activation of N–N bonds and lowering the decomposition energy barrier.

Building upon this, the Cu–O–C interfacial sites formed between CuO and the carbon supports constitute essential auxiliary active sites. The literature [23] indicates that the Cu–O–C interface formed in Cu-based oxide/carbon heterojunctions facilitates the separation and migration of charge carriers, thereby enhancing the catalytic efficiency. Combined with the UPS and XPS characterization results of this study, it can be speculated that the Cu–O–C interfacial sites exert an auxiliary synergistic effect by promoting electron redistribution and reinforcing interfacial coupling. This interface does not supplant the dominant role of the coordinatively unsaturated Cu(II) sites; instead, it enhances the activation capacity of CuO towards azide groups by improving the interfacial charge transfer efficiency, modulating the local electronic state distribution, and stabilizing the electronic environment surrounding the active centers, thus exhibiting catalytic performance superior to that of pure CuO.

The optimal catalytic activity of CuO/CNT originates from the synergistic interplay of particle size and dispersion state, interfacial electron transfer capability, and Cu valence cycling efficiency. First, the morphological and BET characterizations reveal that CuO in CuO/CNT possesses a smaller particle size and a more uniform dispersion, exposing an increased number of surface active sites. Concurrently, its higher specific surface area and pore volume are conducive to gas–solid contact, mass transfer diffusion, and localized heat transfer. Second, the UPS results demonstrate that CuO/CNT has the lowest work function, indicating the highest electron transfer efficiency at the Cu–O–C interface. This is beneficial for enhancing the electron tunability around the active Cu sites and elevating the activation efficiency. Third, the excellent electrical conductivity and high degree of graphitization of CNTs can promote interfacial electron migration and accelerate the redox cycling between Cu^2+^ and Cu^+^, enabling the active centers to complete the catalytic cycle more rapidly and sustain high reactivity.

In summary, the proposed synergistic catalytic mechanism for this system is as follows: the coordinatively unsaturated Cu(II) sites on the highly dispersed CuO surface act as the core active centers dominating the activation of azide groups; the Cu–O–C interfacial sites provide an auxiliary synergistic effect by modulating the interfacial electronic structure and facilitating carrier migration; meanwhile, the defect sites on the carbon supports primarily function to anchor and disperse CuO. Among them, owing to its superior structural dimensionality and electron transport properties, CNT empowers CuO/CNT to manifest the most robust catalytic efficacy under the synergy of multiple factors.

It should be emphasized that the aforementioned mechanistic analysis is primarily predicated on static characterizations and thermal analysis results, representing a rational structure–activity relationship inference. Direct evidence regarding the interfacial charge transfer dynamics, the evolution of active intermediates, and the dynamic variations in Cu valence states during the reaction process awaits further verification through in situ/operando characterization techniques.

## 4. Conclusions

In this work, two carbon-supported catalysts (CuO/GO and CuO/CNT) were prepared, and their effects on the thermal decomposition behavior of GAP-ETPE were compared with that of pure CuO. Furthermore, their structure–activity relationships were elucidated by coupling various structural characterizations with thermal analysis techniques. The results demonstrate that the introduction of carbon supports does not alter the monoclinic crystal phase of CuO, but significantly improves the dispersion state of the active phase and the interfacial characteristics. Compared to pure CuO, both GO and CNT carriers enlarge the specific surface area and increase the number of active sites. Notably, the tubular network, high degree of graphitization, and open pore structure of CNT exhibit superior performance in inhibiting CuO agglomeration, facilitating electron migration, and enhancing mass and heat transfer. Consequently, the CuO/CNT composite demonstrates a more potent synergistic catalytic capability. TG/DTG and DSC analyses indicate that all three catalysts promote the first-stage thermal decomposition of GAP-ETPE, leading to a significant decrease in both the maximum-mass-loss-rate peak temperature and the exothermic peak temperature. Among them, CuO/CNT advances the exothermic decomposition peak to the greatest extent, indicating its most pronounced catalytic effect on the decomposition of the azide groups. The TG-MS results further reveal that the species of the primary gaseous products remain unchanged after the addition of catalysts, indicating that the main thermal decomposition pathway and product distribution pattern of GAP-ETPE are not fundamentally altered. The core role of the catalysts lies in lowering the activation energy barrier for the decomposition of azide groups through interfacial charge transfer, thereby achieving a significant reduction in decomposition temperature and an acceleration of the reaction rate.

Overall, under the preparation conditions in this study and with similar CuO loading levels, the regulatory capability of the three catalysts on thermal decomposition follows the descending order of CuO/CNT > CuO/GO > CuO. The CuO/CNT composite exhibits the optimal synergistic catalytic performance, which is driven by the combined effects of several factors: (1) the structural advantages of CNTs and their specific preparation process lead to smaller CuO crystallite sizes and a more uniform dispersion of active sites, ensuring excellent intra-sample uniformity; (2) the intrinsic tubular network, high degree of graphitization, and open pore structure of CNTs demonstrate superior performance in inhibiting CuO agglomeration, facilitating electron migration, and enhancing mass and heat transfer.

Finally, it should be noted that the discussion of the catalytic mechanism and the structural characterization data in this study are primarily based on ex situ measurements. Although the deduced nitrene intermediate pathway and interfacial charge transfer mechanism are highly consistent with existing literature, direct observation of the dynamic evolutionary behavior of the catalysts during the actual thermal decomposition process—such as real-time changes in the Cu(II)/Cu(I) oxidation states and the generation or consumption of surface-adsorbed intermediates—is still lacking. Moreover, the temporal resolution of the current TG-MS limits the precise capture of transient intermediate lifespans and subtle changes in the competitive proportions of different reaction pathways. In future studies, techniques including in situ diffuse reflectance infrared Fourier transform spectroscopy (in situ DRIFTS), in situ X-ray photoelectron spectroscopy (in situ XPS), electron spin resonance (ESR), and high-temporal-resolution time-of-flight mass spectrometry (TOF-MS) can be utilized to track the real-time evolution of the electronic states at the catalytic active sites and the dynamic behavior of reaction intermediates during thermal decomposition. This will provide more rigorous experimental evidence and design insights for the application of carbon-based composite catalysts in energetic materials.

## Figures and Tables

**Table 1:** Pore structure parameters of the samples.

Sample	Specific Surface Area (m^2^ g^−1^)	Pore Volume (cm^3^ g^−1^)	Average Pore Diameter (nm)
CuO	22.70	0.10	13.85
CuO-GO	48.13	0.22	13.42
CuO-CNT	172.67	0.92	20.05

**Table 2:** Thermal decomposition parameters of GAP-ETPE and GAP-ETPE with three thermal decomposition catalysts.

Sample	*T_p_*_1_ (°C)	W (%)	Residue (%)	*T_p_* (°C)	Δ*H_d_* (J/g)
GAP-ETPE	251.03	31.94	15.23	253.32	1684.63
CuO@ETPE	230.94	29.01	27.85	233.81	1763.96
CuO/GO@ETPE	225.61	30.73	22.11	230.57	1637.51
CuO/CNT@ETPE	217.67	30.22	29.37	224.84	1720.73

*T_p_*_1_ is the temperature of the maximum weight loss rate in the first stage of the DTG curve; W is the weight loss in the first stage; $T_{p}$ is the thermal decomposition temperature in the DSC curve; $\Delta H_{d}$ is the heat of decomposition.

## Data Availability

The original contributions presented in this study are included in the article. Further inquiries can be directed to the corresponding authors.

## Supplementary Materials

The following supporting information can be downloaded at: https://www.mdpi.com/article/10.3390/ma19081542/s1, Figure S1. TEM-EDS elemental mapping images of (a–d) CuO/GO and (e–h) CuO/CNT composites, showing the spatial distribution of C (blue), O (red), and Cu (yellow). Figure S2. The TG of GO, CNT, CuO/GO and CuO/CNT. Figure S3. DSC, α-T, and Kissinger linear fitting plots of the composites at different heating rates: (a–c) GAP-ETPE, (d–f) CuO/GO@GAP-ETEP, (g–i) CuO/CNT@GAP-ETEP. Table S1. The kinetic parameters of different samples. Figure S4. Particle size distribution of different catalysts: (a) CuO, (b) CuO/GO, (c) CuO/CNT.
