# Supplementary material for: Effect of Different Carbon-Supported Catalysts on the Thermal Decomposition of Energetic Thermoplastic Elastomers
Source: Materials (Basel). 2026 Apr 12;19(8):1542. doi: 10.3390/ma19081542 (PMC13118000; doi:10.3390/ma19081542)
Supplement: Supplementary file 1 [file materials-19-01542-s001.zip › materials-4222239-supplementary.pdf]

Effect of Different Carbon-Supported Catalysts on the Thermal Decomposition of Energetic Thermoplastic Elastomers

Supplementary Material

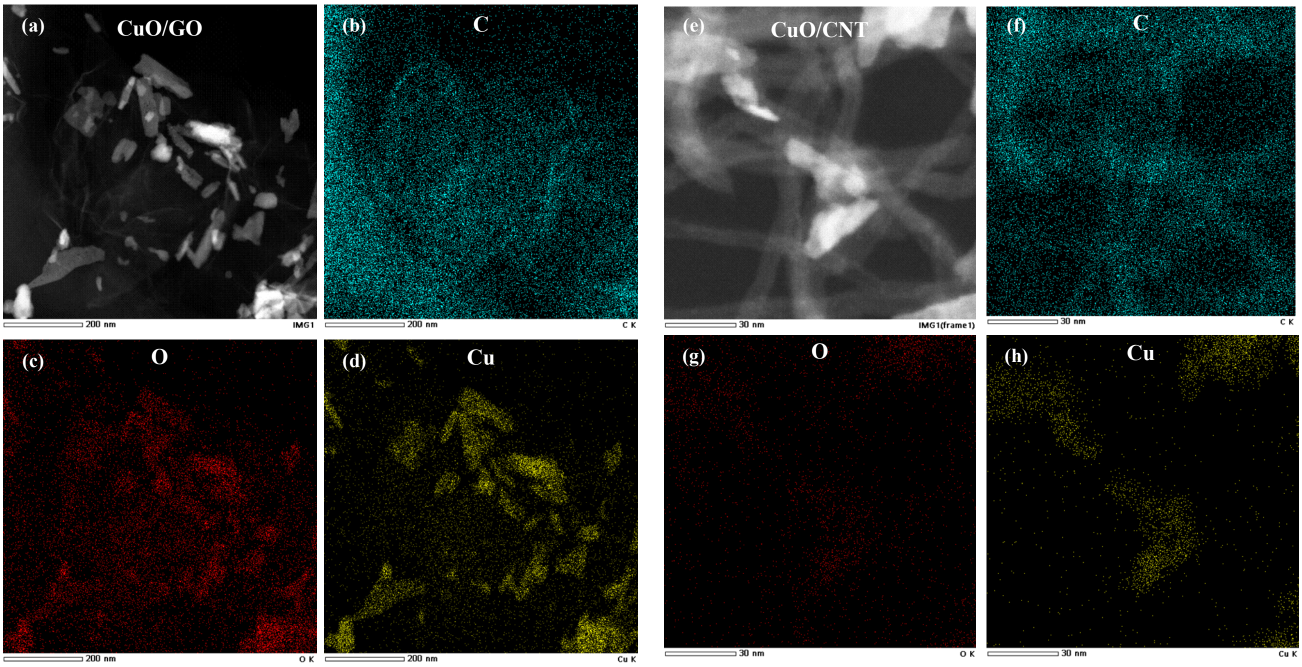

Figure S1.TEM-EDS elemental mapping images of (a – d) CuO/GO and (e – h) CuO/CNT composites, showing the spatial distribution of C (blue), O (red), and Cu (yellow).

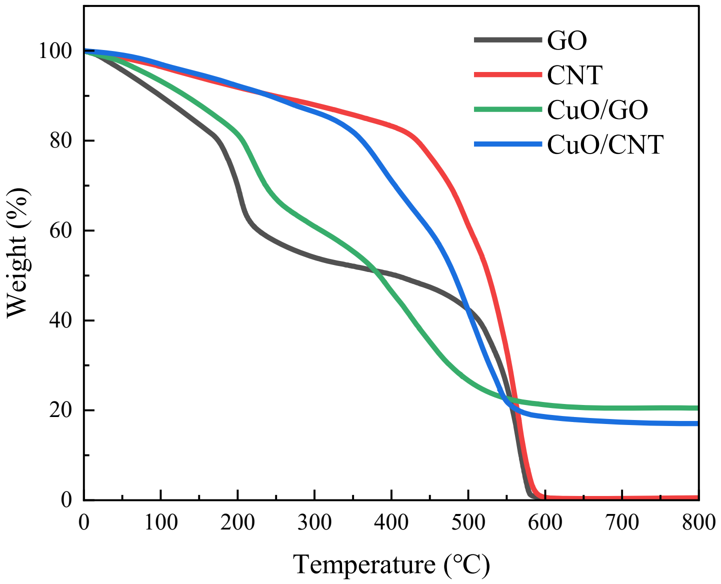

Figure S2. The TG of GO、CNT、CuO/GO and CuO/CNT.

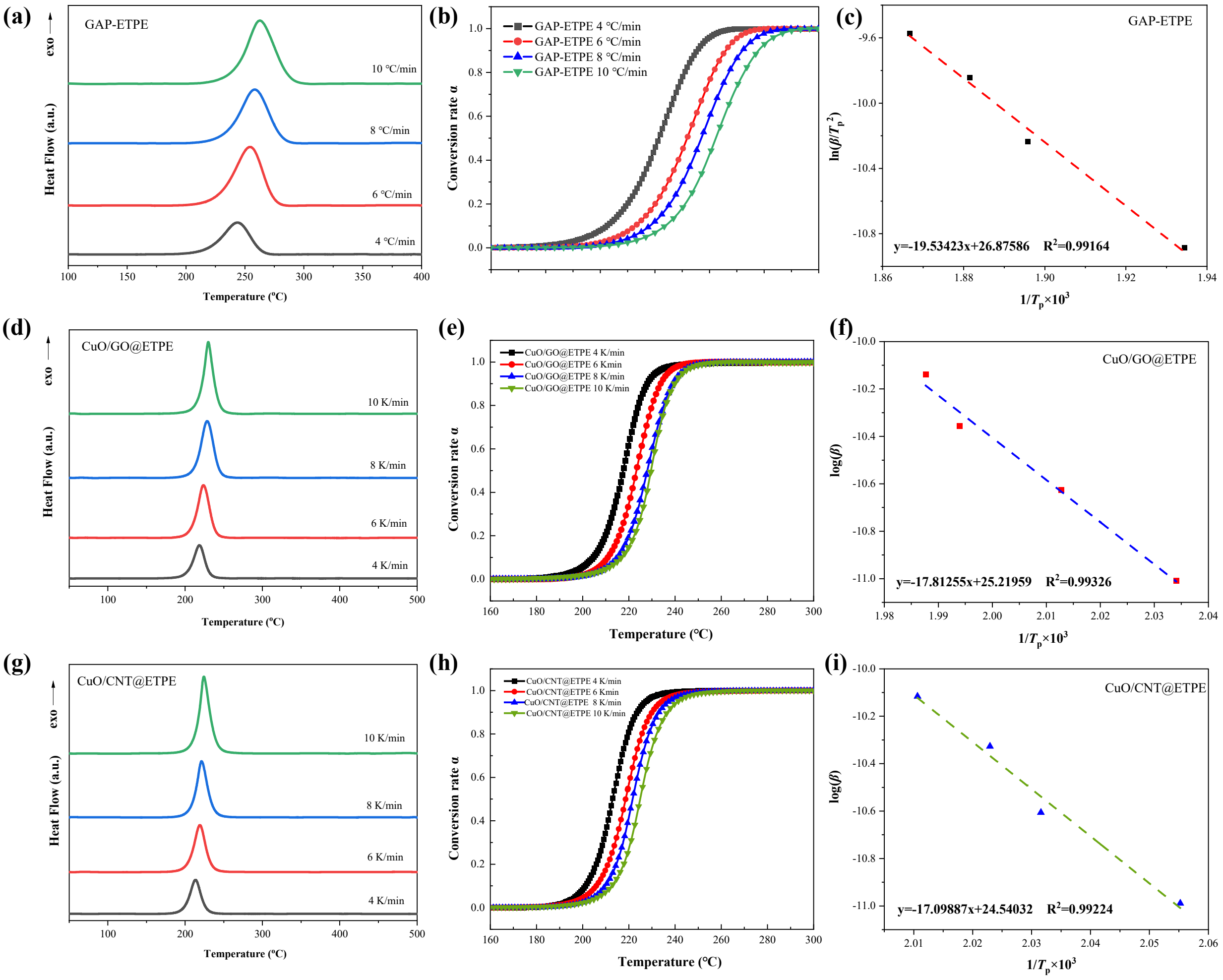

Figure S3. DSC, α-T, and Kissinger linear fitting plots of the composites at different heating rates: (a)–(c) GAP-ETPE, (d)–(f) CuO/GO@GAP-ETEP, (g)–(i) CuO/CNT@GAP-ETEP.

Table S1. The kinetic parameters of different samples

	E_a (kJ/mol)	lnA (s^{-1})	R^2
GAP-ETPE	162.41	36.76	0.99164
CuO/GO@GAP-ETPE	148.09	35.01	0.99326
CuO/CNT@GAP-ETPE	142.17	34.29	0.99224

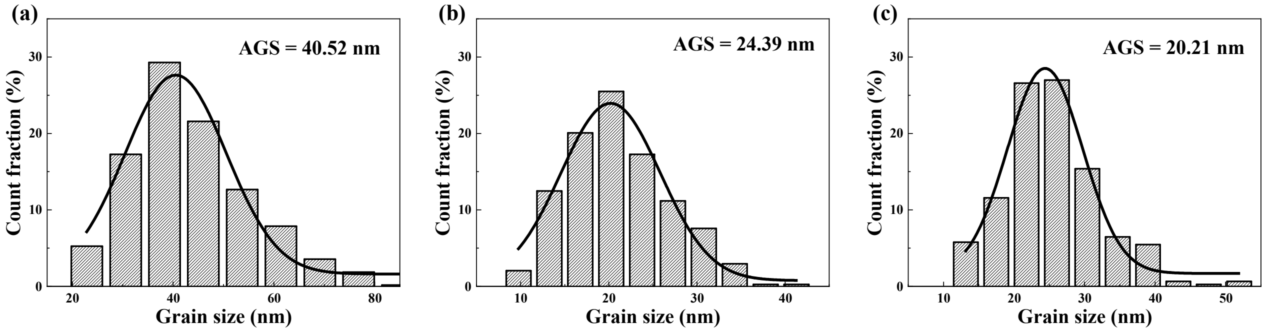

Figure S4. Particle size distribution of different catalysts：(a)CuO, (b) CuO/GO, (c) CuO/CNT.
